# Protein familiarity is a fundamental but rarely operationalized concept in the safety assessment of genetically modified crops: example of phosphomannose isomerase (PMI)

**DOI:** 10.1007/s11248-023-00358-6

**Published:** 2023-07-06

**Authors:** Anne B. Carlson, Carey A. Mathesius, Tim A. Gunderson, Aideen Hession, Reba Bruyere, Henry P. Mirsky, John Zhang, Mat Sandmann, Melissa N. Fallers, Rod A. Herman

**Affiliations:** 1 Corteva Agriscience, 8325 NW 62nd Avenue, Johnston, IA 50131 USA; 2 Corteva Agriscience, Haskell R&D Center, P.O. Box 30, Newark, DE 19714 USA; 3 Corteva Agriscience, 9330 Zionsville Road, Indianapolis, IN 46268 USA

**Keywords:** Phosphomannose isomerase (PMI), History of safe use, Genetically modified, Risk-disproportionate regulation, Negligible risk

## Abstract

Fundamental to the safety assessment of genetically modified (GM) crops is the concept of negligible risk for newly expressed proteins for which there is a history of safe use. Although this simple concept has been stated in international and regional guidance for assessing the risk of newly expressed proteins in GM crops, its full implementation by regulatory authorities has been lacking. As a result, safety studies are often repeated at a significant expenditure of resources by developers, study results are repeatedly reviewed by regulators, and animals are sacrificed needlessly to complete redundant animal toxicity studies. This situation is illustrated using the example of the selectable marker phosphomannose isomerase (PMI) for which familiarity has been established. Reviewed is the history of safe use for PMI and predictable results of newly conducted safety studies including bioinformatic comparisons, resistance to digestion, and acute toxicity that were repeated to gain regulatory reapproval of PMI expressed from constructs in recently developed GM maize. As expected, the results of these newly repeated hazard-identification and characterization studies for PMI indicate negligible risk. PMI expressed in recently developed GM crops provides an opportunity to use the concept of familiarity by regulatory authorities to reduce risk-disproportionate regulation of these new events and lessen the resulting waste of both developer and regulator resources, as well as eliminate unnecessary animal testing. This would also correctly imply that familiar proteins like PMI have negligible risk. Together, such modernization of regulations would benefit society through enabling broader and faster access to needed technologies.

## Introduction

### Familiarity in risk assessment

Fundamental to the safety assessment of genetically modified (GM) crops is the concept of negligible risk for newly expressed proteins for which there is a history of safe use. Proteins with familiarity (safe use) in food and feed should require little or no additional assessment for safety (Roper et al. 2021). This is analogous to the “generally regarded as safe” (GRAS) concept used for food ingredients by the US Food and Drug Administration (FDA) (Burdock and Carabin 2004). In simple terms, food and feed ingredients that have a track record of being safely consumed should be exempt from further regulatory oversight. While this simple concept has been stated in international and regional guidance for assessing the risk of newly expressed proteins in GM crops (EFSA Panel on Genetically Modified Organisms (GMO) 2011; OECD 1986), its full implementation by regulatory authorities has been lacking. As a result, safety studies are continually repeated at a significant expenditure of resources by developers, study results are repeatedly reviewed by regulators, and animals are sacrificed needlessly to complete redundant animal toxicity studies (Garcia-Alonso et al. 2022). Here we illustrate this situation using the example of the selectable marker phosphomannose isomerase (PMI) for which familiarity has been established. The PMI enzyme functions as a selectable marker in tissue culture by enabling plant cells to use mannose as a carbon source (Reed et al. 2001). Reviewed here is the history of safe use for PMI and predictable results of newly conducted safety studies that were repeated to gain regulatory approval of the PMI selectable marker expressed in recently developed GM events in maize. This publication will add to the existing literature on this subject with the hope that regulatory policies will evolve as the evidence becomes overwhelming.

### Familiarity of PMI

The food and feed safety of PMI expressed in GM crops was first described over two decades ago (Reed et al. 2001) and was updated in 2008 (Delaney et al. 2008a). In the latter paper, it is noted that “PMI is ubiquitous in nature” and that PMI enzymes are found in microbes, mammals (including humans), human gut microflora, and less frequently in plants. A recent review concluded there was negligible allergenicity risk for PMI (Herman et al. 2021a). PMI expressed in GM crops has been approved for use in > 25 countries/regions, with over 275 regulatory approvals across these geographies, and maize expressing the PMI protein has been grown commercially in the United States since 2007 (https://www.isaaa.org/gmapprovaldatabase/gene/default.asp?GeneID=37&Gene=pmi). Most recently, “Golden Rice” (high vitamin-A rice; event IR-00GR2E-5) expressing PMI was approved in Australia, Canada, New Zealand, the Philippines, and the United States (https://www.isaaa.org/gmapprovaldatabase/event/default.asp?EventID=528). It seems reasonable to conclude that this level of familiarity with PMI in food and feed without any reports of harm should be considered overwhelming as evidence of safety. As recognition of this, authorities in Argentina recently recognized the appropriateness of reducing the regulatory requirements for familiar newly expressed proteins in GM crops, and included PMI in a list of such proteins (https://www.magyp.gob.ar/sitio/areas/biotecnologia/conabia/_pdf/CIRCULAR_CIyB_N2_HDUS.pdf). However, the results of new safety studies are reported here to facilitate timely approval by regulatory authorities of newly developed GM maize events that express PMI.

## Materials and methods

Production of the PMI protein test substance followed methods summarized in Carlson et al. (2019), Carlson et al. (2022), or Mathesius et al. (2009). Briefly, PMI was produced in an *Escherichia coli* expression system as a fusion protein with a N-terminal His-tag. The tagged protein was purified using Ni–NTA affinity chromatography and the tag was removed with on-column thrombin cleavage. Q Sepharose HP column chromatography was used for further purification followed by thrombin removal using Heparin Sepharose column chromatography. Buffer exchange to 50-mM ammonium bicarbonate was completed using dialysis. The purified protein was then lyophilized and stored in an ultralow freezer until used in the subsequent studies. A set of analyses were conducted to characterize and verify that the microbially produced protein was comparable to the *in planta* version and thus was suitable to use in subsequent safety and characterization studies (Carlson et al. 2019, 2022; Delaney et al. 2008a, b; Gao et al. 2004; Griffin et al. 2013; Harrison et al. 1996; Mathesius et al. 2009). For PMI, this characterization included amino acid composition analysis for concentration, purity analysis using SDS-PAGE/Coomassie staining, western blot for molecular weight and immunoreactivity determination, peptide mapping and intact mass determination by mass spectrometry, N-terminal sequencing and enzymatic activity measurement.

PMI glycosylation status, susceptibility to simulated gastric fluid (SGF), simulated intestinal fluid (SIF), and SGF followed by SIF, and heat stability of the enzymatic activity were determined to further characterize the protein and meet the current expectations of regulatory agencies for allergenicity assessment. A 14-day acute oral toxicity study in mice and in silico bioinformatic analyses of the PMI sequence were also performed to evaluate the potential toxicity of PMI, with the latter also supporting the allergenicity safety assessment. Both the allergenicity and toxicity assessments are foundational for the weight-of-evidence approach used to evaluate safety for newly expressed proteins in GM plants (Codex Alimentarius Commission 2003; Delaney et al. 2008a).

### Characterization of microbially produced PMI

The methods used for concentration determination by amino acid composition analysis, purity by Coomassie staining of the sodium dodecyl sulfate polyacrylamide gel electrophoresis (SDS-PAGE) gels, demonstration of immunoreactivity to a PMI monoclonal antibody and confirmation of molecular weight via western blot analysis, protein identity by liquid chromatography-mass spectrometry (LC–MS) analysis of the trypsin-and-chymotrypsin-digested PMI, molecular mass determination by LC–MS, and N-terminal amino acid sequence determination were previously described in Carlson et al. (2022) and Carlson et al. (2019) are summarized as follows. For amino acid composition, the lyophilized protein samples were solubilized in formic acid and hydrolyzed in hydrochloric acid at a final concentration of 6 N under argon at 110 °C for 24 h, diluted, and then mixed with isotopically labeled amino acid internal standards. Samples and calibration solutions were separated by gradient elution using ultra performance liquid chromatography (ACQUITY UPLC®; Waters Corporation; Milford, MA) fitted with a Cortecs UPLC C18 1.6 µm column (2.1 × 100 mm; Waters Corporation; Milfold, MA). The resulting eluent was directed to the electrospray source on a tandem quadrupole (Xevo TQ™; Waters Corporation; Milford, MA) mass spectrometer operating in the positive mode. Multiple Reaction Monitoring transitions were collected, and quantitation performed using QuanLynx™ software (version 4.1; Waters Corporation; Milford, MA). Molar amounts of each amino acid were used to calculate the protein concentration. For SDS-PAGE, samples were solubilized with 1X LDS sample buffer, heated, diluted further in the same buffer, loaded onto 4–12% Bis–Tris gels (Invitrogen, Waltham, MA) along with molecular weight markers, and electrophoresis performed with a pre-cast gel electrophoresis system. Upon completion of electrophoresis, gels were removed from the gel cassette and used for Coomassie staining, western blot analyses, or sample preparation for peptide mapping and N-terminal amino acid sequencing. The gel intended for Coomassie staining was washed, stained with GelCode Blue stain reagent (Thermo Scientific), destained, and electronically imaged for purity determination using densitometry. For western blot analysis after SDS-PAGE, PMI was transferred to a nitrocellulose membrane using an iBlot Gel Transfer system and the membrane washed and blocked. Sequential incubations were performed using a PMI-specific antibody, a secondary antibody conjugated with horseradish, and a chemiluminescent substrate before capturing the PMI and molecular weight marker images electronically. For peptide mapping following SDS-PAGE, Coomassie staining, and gel imaging, protein bands at the expected molecular weight were excised, then reduced with dithiothreitol, alkylated with iodoacetamide, and digested with trypsin and chymotrypsin. The digested samples were then separated by gradient elution using an UPLC (ACQUITY; Waters Corporation; Milford, MA) fitted with a Cortecs UPLC C18 1.6 μm Column (2.1 × 100 mm; Waters Corporation; Milford, MA). The eluent was directed into an electrospray source operating in positive mode on a hybrid quadrupole-TOF MS (Triple TOF 5600+; Sciex; Farmingham, MA). Data were subsequently processed using MS Data Converter (Beta 1.3; Sciex; Farmingham, MA) and an MS/MS ion search was performed (Mascot Software version 2.7.0; Matrix Science; Boston, MA) to match peptides from the expected PMI sequence (Perkins et al. 1999). GPMAW software (version 12.11.0; Lighthouse Data; Odsense, Denmark) was used to calculate the combined sequence coverage. For intact mass determination, a lyophilized-PMI sample was solubilized and then diluted in 2% acetonitrile/0.1% formic acid solution prior to the mass determination by gradient elution using an UPLC (ACQUITY; Waters Corporation; Milford, MA) fitted with a Acquity UPLC BEH C4, 300 Ǻ, 1.7 μm column (2.1 × 100 mm; Waters Corporation; Milford, MA). The eluent was then directed into an electrospray source operating in the positive mode on a Triple TOF 5600+ MS (Sciex; Farmingham, MA). The generated data were processed using BioPharma View software (version 2.1; Sciex; Farmingham, MA) to produce and intact protein molecular weight. For N-terminal sequence analysis, a gel was incubated in cathode buffer following SDS-PAGE and the proteins were transferred to a sequencing grade PVDF membrane (Millipore Immobilon-P) using the Trans-Blot SD Semi-Dry Electrophoretic Transfer Cell system. Once the transfer process was complete, the membrane was washed, stained with GelCode Blue, and destained with water. Bands were excised and combined into a single sample which was then analyzed using an N-terminal sequencer (Shimadzu PPSQ-51A; Shimadzu; Columbia, MD) where 10 Edman degradation cycles were performed. During each cycle, the N-terminal amino acid was sequentially derivatized with phenylisothiocyanate (PITC), cleaved with trifluoracetic acid, and converted to phylthiohydantoin (PTH)-amino acid, which were identified through chromatography. LabSolutions software (Shimadzu; Columbia, MD) was used to identify the N-terminal sequence.

### Determination of enzymatic activity for the microbially produced PMI

PMI activity was determined using a coupled enzymatic reaction in a 96-well format. The increase in the final product was measured using spectrophotometrically based methods described in Gracy and Noltmann (1968), with some alterations made according to Hu et al. (2016). Sample preparation started with solubilizing lyophilized PMI in water under chilled conditions, followed by determining the PMI concentration using a validated enzyme-linked immunoassay (ELISA). Master mix consisting of M6P, β-NADP, PGI, G6PDH, and 50 mM Tris, pH 7.5 was prepared and dispensed into the wells of a 96-well plate which was then equilibrated at 25° C in a spectrophotometric plate reader (SpectraMax; Molecular Devices; San Jose, CA) for approximately 15 min prior to adding the solubilized PMI further diluted with 50 mM Tris at pH 7.5. Using Kinetic-PathCheck protocol in Softmax Pro (Molecular Devices; San Jose, CA), the loaded plate was returned to the plate reader and the auto-mix feature used to mix the plate once prior to reading every 20 s for 10 min at 340 nm. Calculations to convert the absorbance change to reaction velocity (mOD/min) were performed by SoftMax Pro GxP and the PathCheck feature was used to normalize this value to 1 cm pathlength. The absorbance change at 340 nm (OD/min) was converted to PMI specific activity units (µmol/min/mg) using the β-NADPH at 340 nm millimolar extinction coefficient of 6.22 mM^−1^ cm^−1^ and a 1 cm pathlength.

### Potential toxicity and allergenicity of microbially produced PMI

Bioinformatic comparisons were performed between the PMI amino acid sequence and the 2020 version of the Corteva internal toxin database and the Comprehensive Protein Allergen Resource (COMPARE) 2020 database (January 2020), the latter available at http://comparedatabase.org, respectively, and as described in Carlson et al. (2019, 2022). Toxicity assessment was further evaluated in a 14-day acute mouse study at a target dose of 5000 mg/kg body weight of PMI along with a vehicle control and 5000 mg/kg body weight of bovine serum albumin with the same parameters assessed as those described in Delaney et al. (2008b), Mathesius et al. (2009), Papineni et al. (2017), Carlson et al. (2019, 2022). SGF and SIF analyses used SDS-PAGE and western blot with lyophilized PMI, BSA, and β-lactoglobulin and the same time course as described in Thomas et al. (2004), Carlson et al. (2019, 2022) for SGF and Delaney et al. (2008b) and Mathesius et al. (2009) for SIF. For the sequential SGF/SIF, PMI was incubated for 1 min in SGF as described and then incubated for 0, 0.5, 1, 2, 5, 10, 20, and 30 min in SIF containing pancreatin at pH ~ 7.5 followed by SDS-PAGE. A Pierce Glycoprotein staining kit was used to determine the PMI glycosylation status following the methods outlined in Mathesius et al. (2009), Carlson et al. (2019, 2022). Presence of any glycoproteins appears as magenta bands in the resulting gel. The spectrophotometric enzymatic assay was also used to assess the enzymatic activity in the aliquots heat-treated at 25, 50, 75, or 95 °C as compared with the enzymatic activity in an unheated control aliquot.

## Results

### Results of characterization of microbially produced PMI

Characterization supported the use of the lyophilized PMI protein test substance for use in subsequent studies. Amino acid composition analysis determined that the concentration of the microbially produced PMI protein test substance was 0.85 mg of protein per mg of lyophilized powder (Table 1). After purity adjustment, the protein concentration was 0.68 mg PMI/mg lyophilized powder.

**Table 1:** Concentration determination by amino acid analysis of PMI via LC–MS

Amino acids	Sample 1 (nmol)	Sample 2 (nmol)	Sample 3 (nmol)
Arginine	22.86	21.07	20.83
Asparagine/Aspartic acid	22.40	20.66	20.10
Glutamine/Glutamic acid	22.36	19.87	20.46
Isoleucine	22.06	20.18	20.18
Leucine	21.28	20.03	20.11
Phenylalanine	21.64	20.30	20.20
Proline	21.47	20.12	20.18
Valine	21.82	20.27	20.04
Average (nmol)	21.99	20.31	20.26
Protein concentration of analytical sample (mg/ml)	4.73	4.37	4.36
Protein to powder (mg/mg)	0.82	0.87	0.82
Overall average (mg/mg)	0.85		

**Fig. 1:**
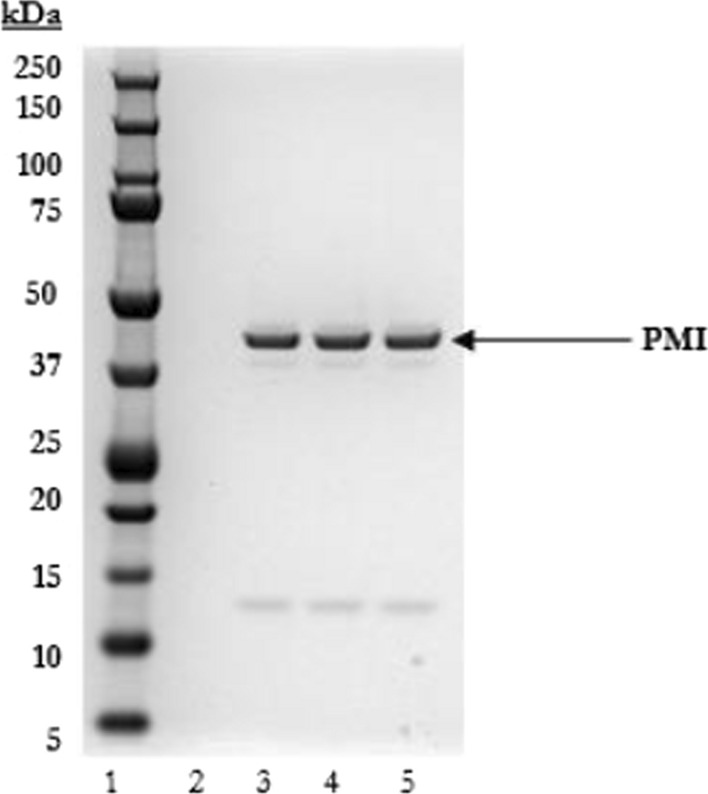
Analysis of the microbially derived PMI by SDS-PAGE (Coomassie staining). Lane 1: pre-stained protein molecular weight markers; Lane 2: buffer only; and Lanes 3 -5 PMI

Coomassie staining of the SDS-PAGE gel (Fig. 1.) demonstrated PMI migrated as a predominant band consistent with the expected molecular weight of approximately 43 kilodaltons (kDa). The purity was determined to be 80% based on densitometry.

**Fig. 2:**
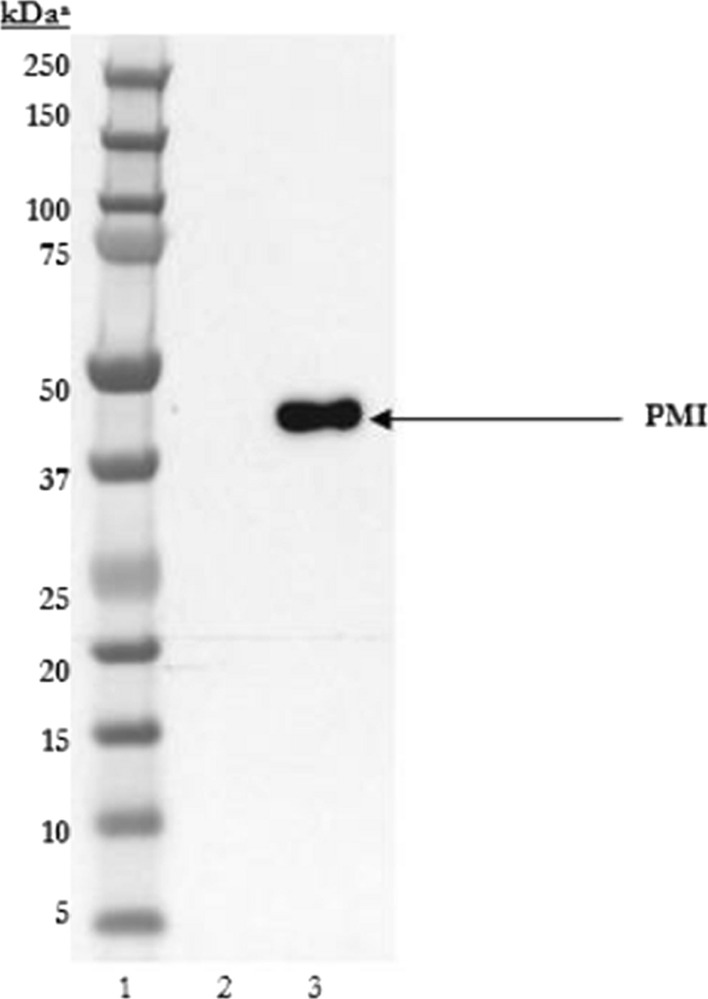
Western blot analysis of microbially derived PMI. Lane 1: pre-stained protein molecular weight marker; Lane 2: buffer only; and Lane 3: PMI

Western blot analysis demonstrated PMI was immunoreactive to a PMI monoclonal antibody and visible as a single band consistent with the expected molecular weight of approximately 43 kDa (Fig. 2.).

The matched peptides identified with the LC–MS analysis of the trypsin-and-chymotrypsin-digested PMI accounted for 92.1% (362/393) of the expected PMI amino acid sequence (Table 2).

**Table 2:** LC–MS analysis identified tryptic and chymotryptic peptides of microbially derived PMI

Matched residue position	Experimental mass^a^	Theoretical mass^b^	Identified peptide sequence
**Trypic peptides**			
6–18	1478.749	1478.752	LINSVQNYAWGSK
19–45	2988.382	2988.382	TALTELYGMENPSSQPMAELWMGAHPK
50–61	1241.670	1241.673	VQNAAGDIVSLR
50–68	2028.047	2028.049	VQNAAGDIVSLRDVIESDK
62–68	804.3834	804.3865	DVIESDK
62–78	1773.938	1773.936	DVIESDKSTLLGEAVAK
69–78	987.5575	987.560	STLLGEAVAK
79–88	1252.696	1252.697	RFGELPFLFK
80–88	1096.593	1096.596	FGELPFLFK
89–104	1773.953	1773.956	VLCAAQPLSIQVHPNK
89–113	2757.437	2757.438	VLCAAQPLSIQVHPNKHNSEIGFAK
105–113	1001.490	1001.493	HNSEIGFAK
114–126	1343.613	1343.614	ENAAGIPMDAAER
182–197	1807.901	1807.903	LSELFASLLNMQGEEK
200–205	627.4302	627.4319	ALAILK
206–220	1714.829	1714.828	SALDSQQGEPWQTIR
283–294	1372.757	1372.76	YIDIPELVANVK
295–309	1682.935	1682.936	FEAKPANQLLTQPVK
357–381	2598.362	2598.365	GSQQLQLKPGESAFIAANESPVTVK
**Chymotrypic peptides**			
1–6	662.3427	662.3421	GSMQKL
7–13	836.3998	836.4028	INSVQNY
14–21	832.4424	832.4443	AWGSKTAL
14–24	1175.615	1175.619	AWGSKTALTEL
16–25	1081.562	1081.566	GSKTALTELY
26–39	1575.669	1575.67	GMENPSSQPMAELW
61–71	1261.651	1261.651	RDVIESDKSTL
61–72	1374.7330	1374.7350	RDVIESDKSTLL
72–80	989.5652	989.5658	LGEAVAKRF
73–80	876.4784	876.4817	GEAVAKRF
97–111	1705.849	1705.854	SIQVHPNKHNSEIGF
112–128	1819.851	1819.852	AKENAAGIPMDAAERNY
129–139	1322.697	1322.698	KDPNHKPELVF
145–153	1113.525	1113.528	LAMNAFREF^c^
151–159	1078.563	1078.566	REFSEIVSL
151–160	1191.648	1191.65	REFSEIVSLL
160–173	1427.766	1427.767	LQPVAGAHPAIAHF
161–173	1314.683	1314.683	QPVAGAHPAIAHF
174–182	1068.553	1068.556	LQQPDAERL
174–185	1397.714	1397.715	LQQPDAERLSEL
175–186	1431.7	1431.699	QQPDAERLSELF
191–201	1261.603	1261.608	NMQGEEKSRAL
202–208	714.4619	714.4639	AILKSAL
202–216	1641.835	1641.836	AILKSALDSQQGEPW
205–216	1344.629	1344.631	KSALDSQQGEPW
209–216	945.3804	945.3828	DSQQGEPW
217–221	629.3849	629.386	QTIRL
217–225	1105.612	1105.613	QTIRLISEF
222–233	1402.628	1402.629	ISEFYPEDSGLF
237–243	797.5352	797.5375	LLNVVKL
239–250	1317.672	1317.675	NVVKLNPGEAMF
239–251	1430.758	1430.759	NVVKLNPGEAMFL
251–259	1047.5	1047.503	LFAETPHAY
252–259	934.4155	934.4185	FAETPHAY
253–259	787.3477	787.3501	AETPHAY
260–275	1671.851	1671.85	LQGVALEVMANSDNVL
261–275	1558.765	1558.766	QGVALEVMANSDNVL
266–275	1090.493	1090.497	EVMANSDNVL
296–304	982.5428	982.5447	EAKPANQLL
305–314	1069.574	1069.577	TQPVKQGAEL
305–323	2115.058	2115.053	TQPVKQGAELDFPIPVDDF
305–325	2333.158	2333.158	TQPVKQGAELDFPIPVDDFAF
326–345	2156.096	2156.096	SLHDLSDKETTISQQSAAIL
346–355	1196.515	1196.517	FCVEGDATLW
356–361	659.3584	659.3602	KGSQQL
356–363	900.5007	900.5029	KGSQQLQL
362–370	975.5004	975.5025	QLKPGESAF

^a^The experimental mass is the uncharged mass calculated from the mass to charge ratio of the observed ion

^b^The theoretical mass is the in silico generated mass that matches closest to the experimental mass

^c^This peptide was modified by methionine oxidation (Oxidation-M)

The predominant mass obtained by LC–MS analysis of the protein was 42,993.74 daltons (Da) consistent with the expected mass of 42,993.63 Da based on the PMI sequence. N-terminal amino acid sequence analysis identified a sequence (GSMQKLINSV) matching amino acid residues 1–10 of the expected protein sequence. Enzymatic activity analysis demonstrated that the PMI specific activity was 154 μmol/min/mg protein.

### Evaluation of toxicity and allergenicity of microbially produced PMI

Results of the search of the PMI sequence against the sequences in the Corteva internal toxin database returned no alignments with an *E*-value ≤ 10^–4^. Results of the search of the PMI sequence against the COMPARE database of known and putative allergen sequences found no alignments with an *E*-value ≤ 10^–4^ that were a length of 80 or greater with a sequence identity of > 35%. One contiguous 8-residue amino acid match (DLSDKETT) was found between the PMI sequence and the sequence of an allergen (a putative alpha-parvalbumin from frog, GenBank Accession CAC83047.1; Hilger et al. 2002). This 8-amino acid match is not considered an indication of allergenic risk. Mitigating factors as described by Herman et al. (2021a) include: (1) PMI has a history of safe use in food; (2) the source organism for PMI as expressed in GM crops is *Escherichia coli* for which no natively expressed allergens are known; (3) the 8-amino-acid contiguous match between PMI and the CAC83047.1 parvalbumin frog allergen has been considered a negligible risk in the past by regulators in the approval of other genetically modified crops; (4) there are no known cross-reactive allergens that share an 8-amino-acid contiguous match without also sharing > 35% identity across an 80 amino acid window; (5) the 8-amino-acid contiguous match between the PMI and the CAC83047.1 parvalbumin frog allergen appears to be outside of known allergen epitopes shared by cross-reactive parvalbumins; (6) and PMI does not share three-dimensional structural features that are critical to the allergenicity of allergenic parvalbumins. These data indicate that no toxicity or allergenicity concern arose from the bioinformatics assessment of PMI. Intragastric exposure of 5000 mg PMI /kg body weight to male and female Crl:CD1(ICR) mice did not result in mortality or other evidence of acute oral toxicity, based on evaluation of body weight, clinical signs, and gross pathology (Table 3). Therefore, the acute oral toxicity tolerant dose and PMI LD_50_ were determined to be greater than 5000 mg/kg body weight.

**Table 3:** Mouse acute oral toxicity study with target dose 5000 mg PMI/kg body weight: Mean mouse body weights (g ± SD)

Treatment	n	Days Relative to Start Date					
		Day 1^a^	Day 2	Day 3	Day 5	Day 8	Day 15
**Males**							
Vehicle	6	31.1 ± 2.2	31.7 ± 2.1	31.8 ± 2.0	32.3 ± 2.4	33.0 ± 2.4	34.7 ± 2.5
BSA	6	31.2 ± 1.7	32.1 ± 2.1	31.1 ± 1.8	31.9 ± 1.8	32.4 ± 1.8	34.8 ± 2.1
PMI	6	31.5 ± 2.2	32.7 ± 1.9	31.6 ± 1.9	31.9 ± 2.2	32.5 ± 2.5	35.3 ± 2.4
**Females**							
Vehicle	6	25.1 ± 1.8	25.3 ± 1.9	24.4 ± 1.8	25.3 ± 2.1	26.1 ± 1.3	28.9 ± 1.8
BSA	6	24.0 ± 2.7	24.8 ± 2.4	24.4 ± 2.6	24.7 ± 2.7	25.2 ± 2.5	28.0 ± 2.4
PMI	6	24.8 ± 2.4	24.9 ± 2.5	24.2 ± 2.4	25.1 ± 2.2	25.7 ± 3.0	27.8 ± 1.9

^a^Fasted weight

**Fig. 3:**
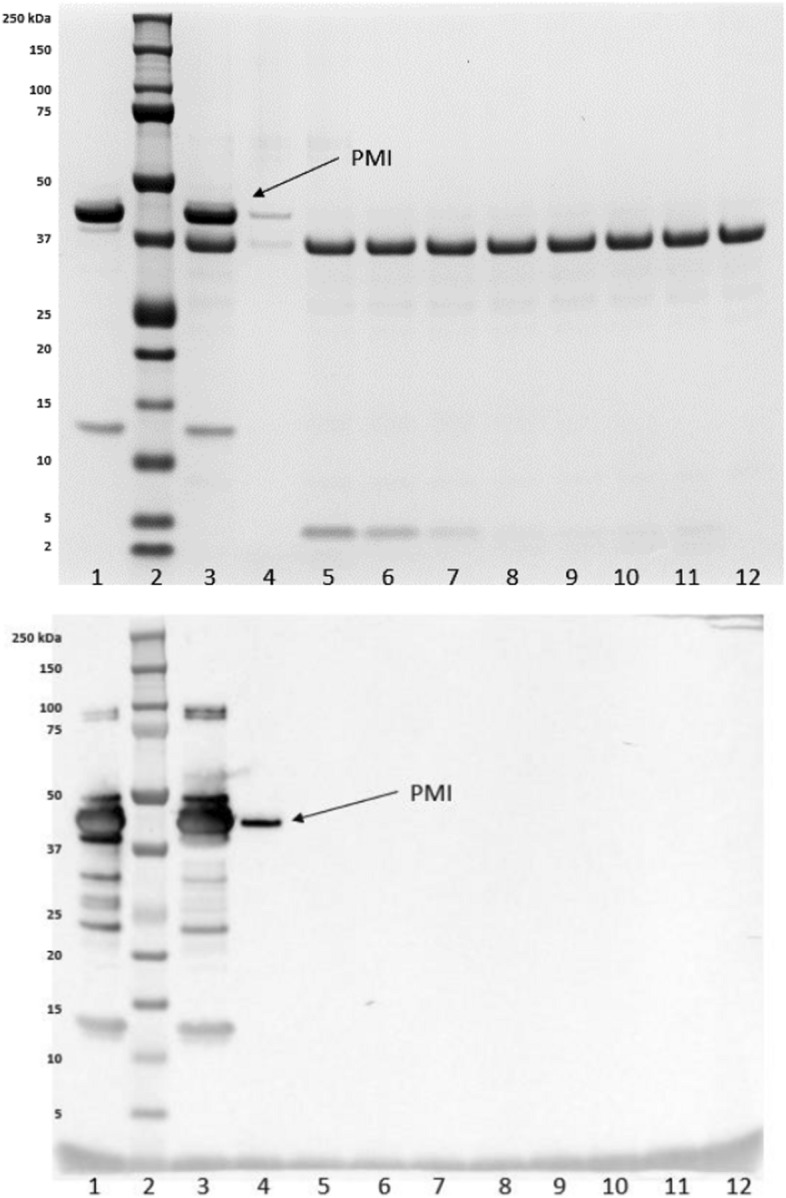
SDS-PAGE analysis (top) and western blot analysis (bottom) of microbially derived PMI from time 0 to 60 min in SGF. Lane 1: PMI in water, time 0; Lane 2: Pre-stained molecular weight makers; Lane 3: PMI in SGF, time 0; Lane 4: PMI in SGF, time 0; 1:20 dilution (SDS-PAGE) and 1:200 dilution (western blot); Lanes 5–11: PMI in SGF for 0.5, 1, 5, 10, 20, and 60 min; Lane 12: SGF control for 60 min

**Fig. 4:**
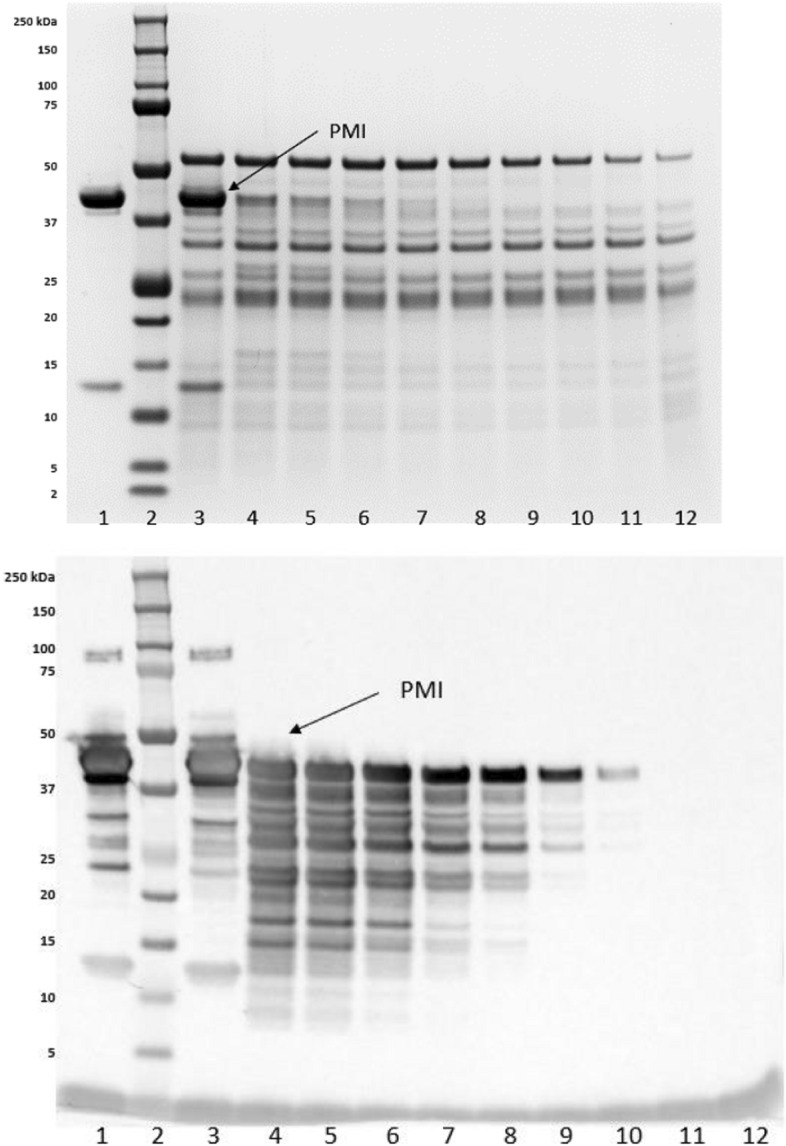
SDS-PAGE analysis (top) and wester blot analysis (bottom) of microbially derived PMI from time 0 to 60 min in SIF. Lane 1: PMI protein in water, time 0; Lane 2: Pre-stained molecular weight makers; Lanes 3–11: PMI in SIF for 0. 0.5, 1, 5, 10, 20, 30, and 60 min; Lane 12: SIF control for 60 min

**Fig. 5:**
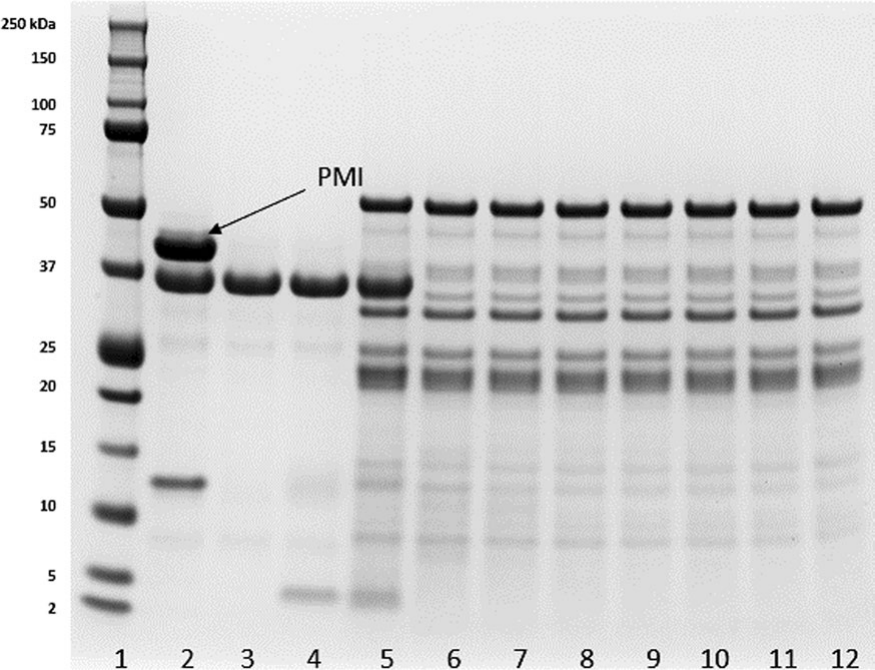
SDS-PAGE analysis of microbially derived PMI in SFG followed by SIF. Lane 1: Pre-stained molecular weight makers; Lane 2: PMI in SGF, time 0; Lane 3: SGF only, 1 min; Lane 4: PMI in SGF, 1 min; Lanes 5–12: PMI in SGF 1 min, SIF for 0, 0.5, 1, 5, 10, 20, and 30 min

**Fig. 6:**
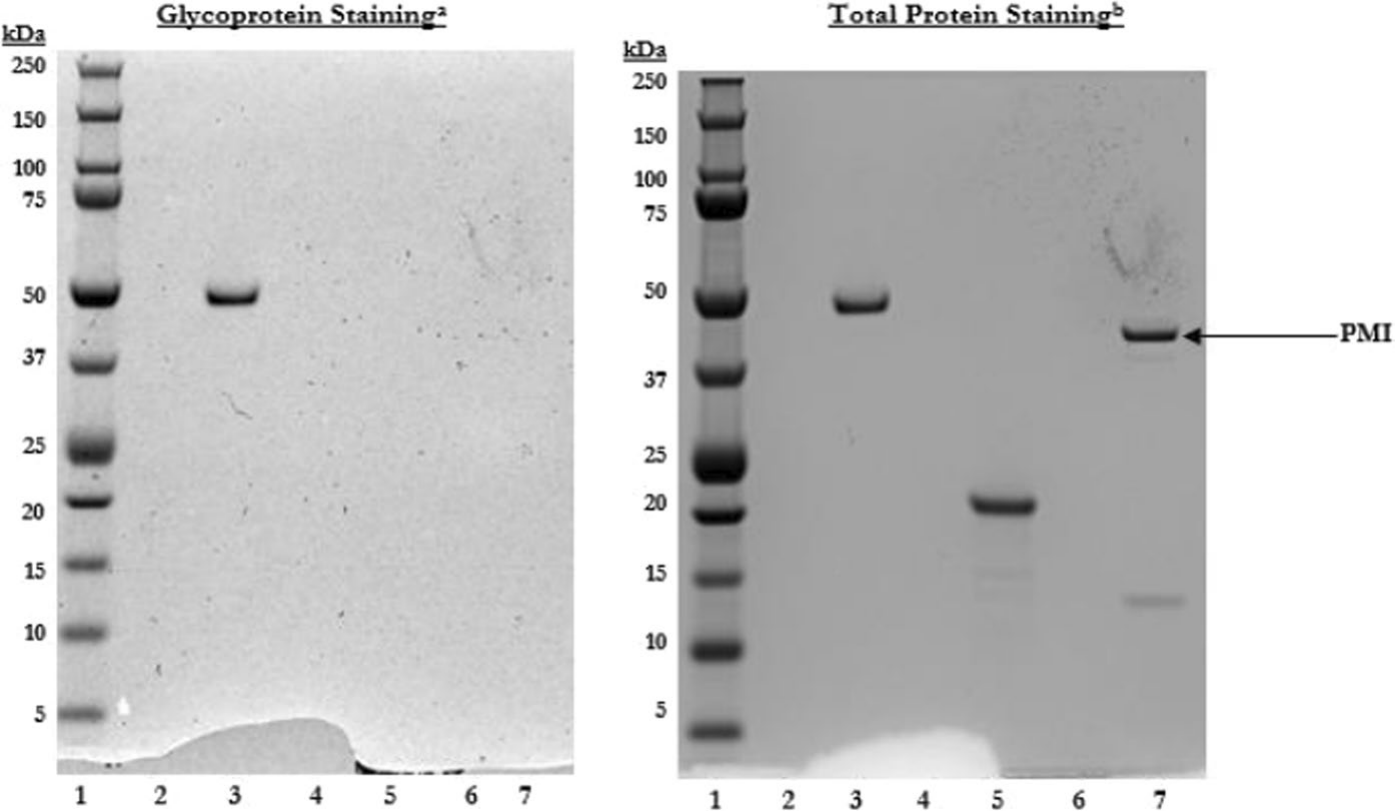
Glycoprotein staining of microbially derived PMI. Lane 1: Pre-stained molecular weight markers; Lanes 2, 4, 6: IX LDS sample buffer; Lane 3: horseradish peroxidase; Lane 5: soybean trypsin inhibitor; Lane 7 PMI. ^a^Gel treated with glycoprotein staining reagent. ^b^Gel treated with glycoprotein staining reagent followed by Coomassie Blue staining reagent for total proteins

The SGF digestion analysis indicated greater than 95% of PMI (the band migrating at ~ 43 kDa) was digested within 0.5 min as shown in both the stained SDS-PAGE gel and western blot. Weak low molecular weight bands (~ 3 kDa) on the SDS-PAGE gel remained detectable in the PMI samples for up to 60 min in SGF (Fig. 3.). In SIF digestion analysis, PMI migrating at ~ 43 kDa was digested within 20 min as shown in the stained SDS-PAGE gel and within 60 min as shown in western blot (Fig. 4). However, the weak low molecular weight bands were digested within 0.5 min in the sequential digestion (Fig. 5.). No protein glycosylation was detected for PMI (Fig. 6.).

Enzymatic analyses results demonstrated PMI was inactivated when heat-treated for 30–35 min at 75 °C and 95 °C, with no activity measured in comparison to the unheated control (Table 4). While heat and digestive stability, as well as the presence of glycans, are typically considered risk factors for allergenicity by regulatory agencies, the current science does not support this belief (Bøgh and Madsen 2016; Goodman et al. 2008; Herman et al. 2022; Privalle et al. 2011; Verhoeckx et al. 2019). However, if a protein were to exhibit a toxic hazard, heat and digestive lability would typically indicate reduced exposure compared with that estimated by expression in the edible tissues of the raw commodity. PMI exhibited no unusual stability to heat or digestion.

**Table 4:** Summary of the effect of heat treatment on PMI enzymatic activity

Treatment	PMI activity units (µmol/min/ml)	% Activity of control
Unheated control solution	219	NA
Test solution heated to 25 °C	220	101
Test solution heated to 50 °C	272	124
Test solution heated to 75 °C	0	0
Test solution heated to 95 °C	0	0

Prior to heat treatment, all treatments were solubilized to a target concentration of 1 mg PMI/ml and diluted. The unheated control solution was maintained chilled (on wet ice). Not applicable (NA).

## Discussion

Predictably, the results of these newly repeated hazard identification and characterization studies for PMI indicate negligible risk since this protein has a history of safe use in food and feed. However, to meet the expectations of current regulations, these studies were repeated in support of new GM maize events expressing PMI. Adding these results to the already substantial scientific literature on this topic will hopefully help facilitate the evolution of government regulations toward more risk-proportional requirements.

It is beneficial to society when the regulation of technology is both proportional to risk and science- based. Although societal acceptance of specific technologies and trust in regulatory decisions is very important in gaining widespread use of scientific advances, conflating efforts to provide the public with information on safety with regulatory oversight can unintentionally cause distrust of a technology because risk-disproportionate regulation implies high risk for low-risk products (Herman et al. 2021b; Strauss and Sax 2016). The incorporation of the concept of history of safe use into the risk assessment and approval process would likely communicate appropriately the negligible risk of GM crops and traits that are familiar. The PMI expressed in recently developed GM maize events and any future events potentially even across crops provides an opportunity to use the concept of familiarity by regulatory authorities to reduce risk-disproportionate regulation of these new events and lessen the resulting waste of both developer and regulator resources, as well as eliminate unnecessary animal testing. This would also correctly imply to the public that familiar traits like PMI have negligible risk. Modernizing regulation to appropriately integrate history of safe use for proteins newly expressed in GM crops into the risk assessment would, in this way, benefit society by enabling useful crop traits to also be developed by academic scientists, non-profits, and smaller entities that help people in both developed and developing countries.
